# Narrative Review: The (Mental) Health Consequences of the Northern Iraq Offensive of ISIS in 2014 for Female Yezidis

**DOI:** 10.3390/ijerph16132435

**Published:** 2019-07-09

**Authors:** Pia Jäger, Claudia Rammelt, Notburga Ott, Angela Brand

**Affiliations:** 1 Section for Social Policy and Social Economy, Faculty of Social Sciences, Ruhr-University Bochum, Universitätsstr. 150, 44801 Bochum, Germany; 2 Department of International Health, Faculty of Health, Medicine and Life Sciences, Maastricht University, Duboisdomein 30, 6229 GT Maastricht, The Netherlands; 3 United Nations University—Maastricht Economic and Social Research Institute on Innovation an Technology (UNU-MERIT), Maastricht University, Boschstraat 24, 6211 AX Maastricht, The Netherlands; 4 Section for Church and Christian History, Faculty of Protestantic Theology, Ruhr-University Bochum, Universitätsstr. 150, 44801 Bochum, Germany

**Keywords:** Yezidi/Yazidi, genocide, ISIS/IS, Northern Iraq offensive, transgenerational trauma, PTSD

## Abstract

The Yezidis who represent a religious minority living in Northern Iraq were particularly affected by the persecution by ISIS (Islamic state of Iraq and Syria, syn.: ISIL—Islamic state of Iraq and the Levant) that gained power after 2013. This paper gives an overview of the events and the mental health consequences on the Yezidi community as well as associated influences on affected female Yezidis. Based on a systematic literature search, the aspects of “Persecution by ISIS and actual situation of the Yezidi community”, “Gender-specific aspects of the persecution and its consequences”, “Mental health of the affected women”, and “Cultural–historical and religious context” are worked out. Research indicates a high burden of health strain and mental health problems in the surviving Yezidi women, especially post-traumatic stress disorders (PTSD) and depression. Concerning transgenerational trauma, the recent genocide has revived past experiences in the history of the community. Like the narrow cultural and religious rules of the community, this can be both a resource and a burden. The actual extent of the attacks is neither predictable for the affected individuals nor for the community, consequences could also be passed onto descendants. Long-term care and support of the affected persons, their descendants, and the Yezidi community seems indispensable.

## 1. Introduction

Inter alia as a result of the civil war in Syria and a political vacuum following the fall of Saddam Hussein, the Islamic terrorist militia ISIS (Islamic state of Iraq and Syria, syn.: ISIL—Islamic state of Iraq and the Levant group) gained power in 2013 [1,2]. Gradually, ISIS brought Iraqi and Syrian areas under its control with the goal to establish an Islamic caliphate based on the fundamental Islamic “God’s law” Shari’a [3,4]. One of ISIS’ most prominent offensives was the so-called “Northern Iraq offensive” in August 2014. Here, ISIS brought a large part of the Northern Iraqi areas, including the region “Sinjar”, under its control. Due to the cruelty of the action this offensive is also known as the “Sinjar massacre” [5].

Both Muslims, who did not share ISIS’ understanding of Islam, as well as Christians and members of other religious minorities experienced displacement, persecution, or were forced to convert. Particularly affected, however, were the Yezidis as they were exposed to persistent rumors and prejudices such as “devil worshippers” and considered to be “infidels” by ISIS [6,7,8,9,10]. Depending on gender, male and female Yezidis had different experiences with ISIS. 

The aim of this paper is to give an overview of the events and the mental health consequences as well as associated influences on affected female Yezidis in Northern Iraq.

## 2. Methods

The methods of this narrative review were planned according to the requirements of Cochrane [11] and PRISMA-P [12,13].

### 2.1. Electronic Literature Search in Scientific Databases

A systematic literature research with the databases “pubmed”, “Cochrane”, “Dimdi”, and “Google Scholar” was done. Concerning the mental health of displaced Yezidi, all research articles published after 2014 were taken into account. Regarding the cultural–historical and religious context, all research articles were considered without limitations of date. Due to the different spellings and authors named “Yazidi” etc., the terms “Yezidi”, “Yazidi”, and “Ezidi” in title and summary were used in the search.

The following Table 1 gives an overview of the results of the literature search.

### 2.2. Selection and Inclusion Criteria

From the results found in this research, the studies dealing with the following subjects were included:

▪ Persecution by ISIS and actual situation of the Yezidi community;
▪ Gender-specific aspects of the persecution and its consequences;
▪ (Mental) Health of the affected women;
▪ Cultural–historical and religious context.

All studies that focused on Yezidi women or Yezidi people (women and men) were included in general. Studies that only dealt with men or children were excluded.

Both qualitative and epidemiological surveys were considered if a standardized scientific survey took place. If there was no standardized survey design, the studies were excluded or used in the “gray literature”.

With respect to the cultural–historical context, publications with a systematic overview of relevant aspects were included. Publications that only focus on specific aspects and those that do not adequately substantiate their sources and, therefore, do not contribute to classifying the current context in terms of cultural or religious history were excluded, too.

Publications with a clear conflict of interest or strong political orientation were not considered. Especially with regard to gray literature, attempts were made to use reports and articles as neutral and trustworthy sources or press.

Language should not be an exclusion criterion. For the use of foreign-language articles that are not available in English, translation software was used or, if necessary, interpreters were consulted.

### 2.3. Gray Literature

Concerning the actual situation, the gender-specific aspects and the cultural–historical context, gray literature, such as reports of national and international organizations and local and international press, as well as literature from the personal collection of the authors, were additionally taken into account.

### 2.4. Handsearching

As additional sources, books and articles from the personal collection of the participating authors were supplemented.

## 3. Results

**Figure 1:**
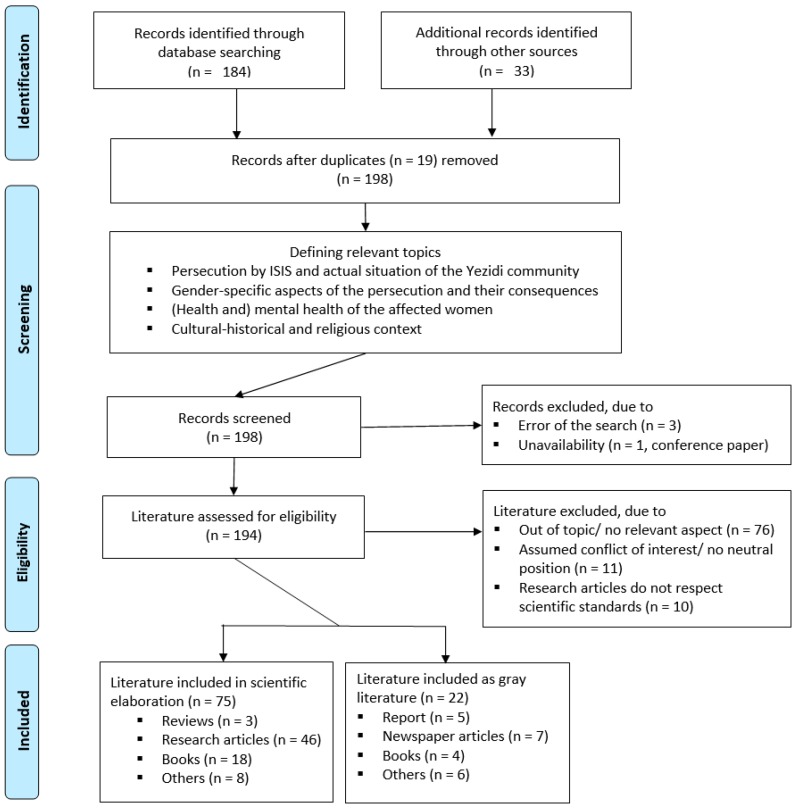
Flow diagram for the selection of literature.

The process of selection of relevant literature based on the guidelines of PRISMA is presented in the following flow diagram (Figure 1) [14].

After consideration of the topic, selection criteria, and the exclusion of duplications as described above, the following literature selection resulted (Table 2):

The literature used from the systematic search as well as the gray literature and additional sources are listed as a table in the Supplementary Materials (see Table S1).

Based on the systematic compilation of literature, a differentiated presentation of the research subject was made. The categories “Persecution by ISIS and actual situation of the Yezidi community”, “Gender-specific aspects of the persecution and their consequences”, (Mental) Health of the affected women”, and “Cultural–historical and religious context” form the lead structure of the literature assignment, whereby individual contents can also be used across.

### 3.1. Persecution by ISIS and Actual Situation of the Yezidi Community

Even after the so-called “Sinjar massacre”, violence and persecution against Yezidis was rampant. Until now, Yezidi people are kidnapped or killed [15]. Still, there is a high number of Yezidi women in ISIS’ captivity. Estimates presume that in 2017, 2500 Yezidis (male and female) [8] or 3000 Yezidi women and children are still in the hands of ISIS [16]. Other sources report that at least 6000 Yezidi women have spent some time in ISIS imprisonment [17]. The atrocities committed by ISIS can be called a systematic destruction of the Yezidi community [18] that shakes the Yezidi society over a long term [19].

The violence was not only directed against members of the religious communities, sacred sites and temples were systematically ruined, as well. At the same time, the local religious roots of Yezidis living in exile/diaspora were destroyed. By this “religious cleansing” of persons and physical structures, ISIS seeks to create a homogenous neo-Salafi space [20].

On 16 June, 2016, the “UN Commission of Inquiry on Syria of the UN Human Rights Council” confirmed that the actions of ISIS against the Yezidis in Northern Iraq constituted a genocide that not only took place in the days of the “Sinjar massacre” but still continues. The report cites the statement of the Chairman of the Inquiry Committee, Paulo Pinheiro: “*Genocide has occurred and is ongoing. ISIS has subjected every Yazidi woman, child or man that it has captured to the most horrific of atrocities*“ [21]. A legal review and prosecution of the genocide crimes against the Yezidis has begun only recently [22].

Not least because of the public relations of Yezidi associations and survivors, the fate of the Yezidis certainly has international support possibilities, such as the support of the Peshmerga army and PKK for the liberation of the Sinjar [23,24,25]. Nevertheless, the response to the genocide and the protection and assistance provided by the international community are considered insufficient by observers [26].

In 2017, large parts of the areas occupied by ISIS could be reconquered. While some of the displaced Yezidis returned to their home villages [27], many others were unable to do so. Either due to the extent of the destruction or fear of a return and renewed attacks by ISIS, a large number of displaced persons still live as “internally displaced persons” (IDP) in so-called IDP camps. Other Yezidi live as asylum seekers in foreign countries [28], especially in Germany [29] or Turkey [30]. Further, some Yezidi people cannot (or do not want to) return to their homeland after they were forced to convert to Islam [15]. In addition, the relationship with the resident Muslim population—some of whom have cooperated with ISIS—is tense; the shaken trust between former neighbors [31] makes a return difficult.

The situation in the IDP camps in Iraq as well as in the refugee camps is quite precarious, especially in Turkey, both on the supply and legal level.

Regarding Yezidi women in a Turkish refugee camp, it could be shown that there were restrictions concerning regular and healthy food. The conditions in camps were unhygienic. The educational, social, cultural, and religious needs were met insufficiently. Moreover, the coherence to the International Refugee Law is questionable [30,32].

Surveys which were carried out in Northern Iraq describe a lack of supply and care due to remoteness, bad and poor connection, and material poverty in the IDP camps. Until the time of the survey in 2017, the displaced people were provisionally housed in tents or living containers. These offered barely protection to their inhabitants in the searing heat. Laundry facilities were not available. Community toilets were available as toilets; their hygienic condition was completely desolate. Diarrheal diseases were widespread. The standard of medical care was quite below those of the autochthonous population in municipalities. Within the IDP camps, there was a lack of available physicians and medication as well as diagnostic and therapeutic measures. As a result, medical treatment could only take place limited and with a high complication rate. The missing of religious rituals and living habits of the accommodated Yezidi showed clearly that they did not recognize the camps as their “home” [33,34].

Educational access for the Yezidi has also been limited in the past. On the one hand, material poverty made it necessary for children to become involved in agriculture instead of visiting a school. On the other hand, the remote location of some Yezidi villages made it difficult for parents to allow their children to attend school. In this context, it must be taken into consideration, that written language did not play a role in the everyday life of many Yezidi families until a few years ago. The Yezidi tradition is an oral one. Historically, Yezidis have been a “non literate, and even anti literate, community”, with “reading and writing forbidden by Yezidi law to all but a particular dynasty of Sheikhs”. It is estimated that Yezidis began attending school en masse only from around the 1950s onwards. The lack of having attended school on their own might be an additional barrier for some Yezidi parents. Additionally, Yezidi students experience discrimination in the Iraqi education system [6,35].

In 2003/2004 and 2013/2014, it continued to deteriorate. Due to the political instability, the Iraqi educational system made massive losses. Displaced Yezidis have been particularly affected because some IDP camps did not have a school immediately. The hidden settlements as well as shelters in which some of the Yezidi families were accommodated first did not have access to a school at all. Meanwhile, all children in Iraq have the right of visiting a school without school fees. Nevertheless, the transport of the children to their schools is such an expensive and complicated task that it represents a major barrier for several families accommodated in remote IDP camps that do not have a school within the camp [35].

All in all, the community of the Yezidis living in Sinjar, with their strong local connections, has been scattered. With its members divided into returnees, refugees, diaspora, and IDP, the future of these people is uncertain.

### 3.2. Gender-Specific Aspects

“*Although all women are subject in some manner to discrimination based on gender, this is compounded for some women when gender discrimination ‘intersects’ with discrimination on other grounds. Refugee women suffer from both the internalized and external consequences of their often already marginalized identities as women and ethnic, national or racial minorities*” [36].

The experiences of affected Yezidis with ISIS are often gender specific. Although Cetorelli’s research has shown that about the same number of male and female Yezidi have been killed or abducted by ISIS [8], the experiences of survivors vary significantly.

Particularly women and girls are affected by sexual violence. This is systematically used as a “war tool” by ISIS—on the one hand because of the physical and psychological consequences for those affected, on the other hand also because of the consequences for the community [37,38,39,40,41,42].

Thus, it is assumed that in the context of genocide, violence, and, above all, sexual violence, the life force of a community is harmed by destroying its intimate relations on a family level as well as its reproductive force on a community level. Such attacks are supposed to have a strong symbolic and metaphysical component with a destructive force capable of conquering entire groups [43,44]. 

Furthermore, some ISIS fighters seem to believe that if they rape Yezidi women it would make them Muslim [45]. Additionally, ISIS uses sex to motivate the young people to join them [46].

Consistently, various sources and statements of those affected report about mass rapes during the attacks by ISIS, sex slavery (also known as “Sabaya”), and forced prostitution as well as trafficking the abducted young and female Yezidi especially [47,48,49,50].

ISIS has developed a system for the enslavement of Yezidi women: Organizing the kidnapping, transportation, and accommodation of the prisoners. The abducted women were also increasingly “registered”. This complicated an escape and expanded the “trade” and the resulting economic profit for ISIS [17,51]. The “trade” took place between ISIS members as well as urban markets or online auctions [52].

In contrast, abducted men were forced to convert or be killed; Yezidi boys were trained and used as child soldiers. Many men and boys are still missing [53,54,55].

Some researches criticize the current media focus on Yezidi women and the sexual violence they experienced by ISIS. They fear that this focus burdens the consideration of the consequences of persecution and displacement by ISIS for the entire Yezidi community. Additionally, they criticize a resulting victimization which does not do justice to the complex role of the affected Yezidi women [56,57].

The Yezidi woman Nadia Murad, who has been appointed “UN Goodwill Ambassador for the Dignity of Survivors of Human Trafficking” [58] and who herself was abducted and detained as a sex slave writes: “*The Islamists knew how devastating that was for an unmarried Yezidi woman. Our worst fears—those of our community and our clergy, so they will not be resumed—have been shamelessly exploited.”* [59]. In addition, some of the former victims fear that the capturers will come back to retake them as prisoners and also that the ISIS members will escape punishment and go underground [17].

At the same time, some of the affected women are enormously strong. Since the Yezidi women are not only the majority of the Yezidi community at all but also primarily responsible for the parenting, they are instrumental in the transmission of cultural heritage. Therefore, culture is considered to be a composition of *“sets of collective memories, a concept which acknowledges the aspect of culture that consists of shared ideas and beliefs of history ancestry and of life sustained in a community of individuals’ memory, lived, signified, expressed and enacted, which gives heritage and cultural practices their meaning”.* In this way, the Yezidi women can both ‘save’ the Yezidi culture and challenge stigmas that preceded the conflict. To support the activism of affected female Yezidi, gender-specific legal work is required [60,61].

Law enforcement can take place at both the national and international levels through International Criminal Court (ICC) [62].

### 3.3. (Health and) Mental Health of Yezidi Women

As a result of the events of 2014, research has focused on the health and mental health state of the surviving (female) Yezidis.

Recent research points to an increased health strain to this population. In a major household study of displaced people, most of them Yezidi, in Northern Iraq, Cetorelli et al. found a high prevalence of non-communicable diseases: 19.4% had hypertension, 13.5% musculoskeletal conditions, 9.7% diabetes, and 6.3% cardiovascular diseases [63]. All these diseases are associated with increased stress levels [64]. In addition, a high rate of adaptive problems such as psychosomatic and trauma-related symptoms was found in Yezidi genocide survivors [65]. Those affected often do not understand the reason for their condition and cannot assign the symptoms to their understanding of illness [54].

In another study by these authors, a high two-week prevalence, which means the presence or occurrence of diseases during this period, of infectious diseases was found; 17.6% had a respiratory infection during this period, 13.5% had diarrhea, and 12.9% had cystitis. In addition, 9.0% reported about mental problems and 6.2% about chronic diseases [28].

The German Special Quota Program “Sonderkontingent Baden-Württemberg (SK BaWü)—Vulnerable Women and Children from Northern Iraq” offered 1100 Yezidi women in 2015 from Northern Iraq, such as Nadia Murad, the opportunity to come to Germany for their security and psychological treatment [66]. As inclusion criteria for participating, the project manager Prof. Kizilhan describes that the Yezidi women from the area of Sinjar in Northern Iraq were victims of rape and held in captivity for at least three months by the so-called “Islamic State”. They have come to Germany between six and eight months after their captivity [67].

The women chosen to join this program in Germany showed a high prevalence of mental disorders in the first examination carried out in Northern Iraq: 78.1 were diagnosed with PTSD, 63.0% with depression, and 2.7% with an adjustment disorder [66]. In Germany, these women showed more symptoms of adjustment disorder rather than classic PTSD [68].

In this context, it should be noted that symptoms of traumatic disorders are often delayed. Especially in migration, overcompensation is often observed along with initial stabilization. A phase of decompensation with mourning and trauma processing often only begins when everyday life has become established and the feeling of security increases. This delayed course was observed in many cases among the Yezidi women, although some of them had shown strong symptoms since their arrival [66].

In a follow-up of 296 of these Yezidi women after a few months, 67% suffered from somatoform disorder, 53% from depression, 39% from anxiety, and 28% from dissociation. The prevalence of PTSD was 57%; the rate increased with the number of experienced rape events [69].

Research that examined the stress level, found high values in the recorded perceived stress in Yezidi women in IDP camps in Northern Iraq based on standardized testing. Simultaneously, the affected women suffered from poor health state, poor health related quality of life, and a high rate of experienced traumas. In this survey, this self-assessment was congruent with the results of the examining medical doctors who found a high prevalence of diseases requiring treatment related to mental stress [33].

Evaluating the occurrence of PTSD and complex PTSD in female Yezidi who were victims of sexual violence living in post-ISIS camps in Northern Iraq, Hoffman et al. found a prevalence of 50.9% for complex PTSD and 20.0% for PTSD [70]. The authors assume that a complex PTSD may be triggered by post-ISIS camp stress.

Also in a study which was carried out in Turkish refugee camps, it could be shown that Yezidi refugee women suffered from psychological health problems depending on war trauma while they had additionally common problems such as unemployment and poverty [32].

Another study found a frequency of PTSD in 42.9% and of major depression in 39.5% of the Yezidi refugees in Turkish refugee camps. Both disorders occurred more often in women than in men with overall 26.4% having suffered from both disorders. Women with PTSD reported flashbacks, hypervigilance, and intense psychological distress due to reminders of trauma more frequently than men. Men with PTSD reported feelings of detachment or estrangement from others more frequently than women. More depressive women than men reported feelings of guilt or worthlessness [71].

In a study with 416 Yezidi women and girls (65 of whom had survived sexual enslavement) in IDP camps in Northern Iraq, more than 80%, and almost all participants who were formerly enslaved, fulfilled criteria for a probable PTSD. Trauma exposure and enslavement predicted poor mental health. In addition, social rejection in the community of formerly enslaved girls and women mediated the relationship between traumatic enslavement events and depression symptoms [72].

The existing patriarchic and gender roles in the society can be an additional load factor for the Yezidi women [32]. High moral conceptions, limitations, and internalized attitudes concerning ‘honor’ and the ‘violation of honor’ can lead to considerable worry and the fear of collective exclusion [54]. In a qualitative approach, a Yezidi woman, in referring to the women who had been abducted and raped by ISIS, said: “*They dishonored our women. Even if they return, they return dishonored (she means their pregnancy)”* [73].

There are records about even less acceptance for women who become pregnant by rape. The deeply engrained culture of the Yezidi prefers that the women do not keep the ISIS fathered babies. If they keep the child, they live outside the traditional parameters of the community and the child will not be accepted. In addition, they have to fear not being supported. Thus, a high number of illegal abortions, partly enforced by the community, took place [41,74,75,76].

However, as a result of mass rape, the Yezidi community has established special rituals in its holy city Lalish to rehabilitate the abducted women. Although it is still believed that there is a danger of being discriminated against by the own community, and some Yezidi women have committed suicide—inter alia because of this fear—after having experienced rape, so far no cases of “repudiation” are officially known. “They are still our children”, the high priest of the Yezidis supposedly said [6,46,77,78].

While on the one hand these social context structures can be a burdening factor, specific resources result on the other hand.

In addition to internal coping strategies, external strategies can also be used for trauma processing: Some women attempted to find meaning in the massacre. It strengthened them in their intention to be strong people who would not give up their religion; they sometimes became more politicized and engaged in active self-defense in order to protect themselves from further possible massacres. They were more committed to religious and cultural rituals as a way to strengthen solidarity among the Yezidi community. The community acceptance and “silent support” of the women who were exposed to sexual attacks may thus express an external coping strategy [73].

To support the affected women and create a basis for rehabilitation, healing, and empowerment, an intensive psychologic, organizational, and material support of the surviving women, including security, protection, and help to open up and verbalize the experience, is required [79]. However, it should be noted that even within the established camps, refugee women are more likely to face discrimination and gender-specific violence [30].

In 2015 and 2016, 1100 women, most of them Yezidi, were sent to a special quota project in Germany for trauma therapy and rehabilitation. While on the one hand such initiatives can offer required treatment possibilities [66], it must not be forgotten that those affected in the diaspora, on the other hand, remain attached to, and are empowered by, a “home” culture of fundamental values like propriety and religion [80].

There may also be difficulties in receiving psychotherapeutic measures: Despite increasing acceptance, only 16.4% of women participating in the program were in psychological treatment and 5.5% already completed one after two years [66]. Moreover, it is criticized that the explicit exclusion of all adult male family members is against the wishes of some women survivors and may compound a lack of agency to determine the conditions of their own recovery and future [81].

### 3.4. Cultural–Historical and Religious Context

“*The Yezidis are a minority in a double sense: As a Kurdish subgroup, they share the fate of the Kurds, who have been persecuted and oppressed for centuries; In addition, as a religious minority, they were often discriminated against by the Kurdish majority religion, Islam.*” [82].

The Yezidis (syn.: Yazidis, Ezidis) understand themselves as a monotheistic religion belonging to the region “Sinjar of the governorate Nineveh” which is their traditional main settlement before Islam was established [83].

It would be too simplistic to regard the religious community as a homogenous group [82]. Furthermore, before 2003, there have been controversial views within the community regarding the Iraqi understanding of nationality [84] as well as a self-perceived belonging to the Kurds [85].

The history of the faith community, especially the pre-modern history, is difficult to reconstruct, especially because there were no written sources until the mid-20th century [86,87,88]. Researchers observe a common substratum with Zoroastrian and Yarisan traditions that probably go back to Indo-Iranian or local traditions [89]. This assumption is not undisputed [90]. However, the religious background of the community is not certainly known.

Over time, Yezidism has incorporated a number of elements of Gnosticism and Oriental Mysticism [91], such as the presence of several “holy beings” [92] as well as of the later Christian and Islamic faith history, so that the Yezidi now represent a belief system which reflects a syncretism of various cults of the area [93,94]. As a result, the Yezidi religion has a “flexible and adaptable belief system” and no fixed dogmatic-theological categories and systems [95]. Thus, the existing religious texts are not normative works [96].

In this context, it can be spoken of as an “oral tradition” of the Yezidi, in which (lyrical) songs and prose narrative play an important role. Only within the last two centuries, and especially within the last decades, has an increase in the importance of written sources been recorded [97,98,99,100].

In the Yezidi belief system, the one and only god is fundamental; furthermore, the seven archangels play an important role. The reformer Sheik Adi introduced a cast system including “Sheikhs” (highest religious authority), “Pirs” (saints, priests), and “Murids” (laymen, disciples) in the 11th century. After that every member of the community has a specific role in the social hierarchy [95,101,102].

Within the strict endogamy of this closed society, marriages between the casts and different religious communities is forbidden [103]. Compliance with these rules is a prerequisite to enter paradise according to the Yezidi faith [104].

The events of massive attacks by the IS are also to be considered in a historical context: The history of the Yezidis is characterized by recurring phases of oppression and attacks by Muslim or Ottoman rulers. Thus, historical resources record 74 episodes of genocide [40,44,46,105,106,107]. Thus, Yezidis are facing three types of trauma: Their individual recent trauma, a historical trauma, and a collective trauma as well [45].

These recurrent experiences of oppression and persecution are deeply rooted in the collective memory of the community and are reflected in songs, stories, and transmissions [10]. Even the exiled Yezidi community is affected by the fate of their faith brothers who revive these individual, collective, and generational memories [44]. While guilt, shame, and depression are frequent as a result of the genocide in the Yezidi population, the recurrent experiences have led to a determined, long-standing ethnic pride, which is now available to those affected as a resource [108].

Initial research indicates that in response to the attempted genocide by ISIS and the situation of displacement, cultural attitudes are changing. These changes affect every-day traditions and behavior [34] and attitudes towards toward suicide as well as rape and pregnancy as a result of rape and doubts about the religion [109]. On the other hand, religious and ritual life has become more intense for some affected rather than declining—probably because of the peoples’ need for supernatural help, new opportunities, and a sense of defiance [110].

The increasing proportion of the community living in the diaspora also leads to a transformation of religion. Thus, Yezidis, especially intellectuals, have begun to construct and theologize the religion of the Yezidis [111,112]. Many, especially older members, have the fear of „dying out” [6], because the younger generation of Yezidi living in Europe increasingly questions traditions and rules [111].

## 4. Discussion

In the context of the Yezidi genocide a transgenerational trauma must also be assumed among the community; the renewed experiences of ISIS are thus a resurgence of a deeply rooted violation of societal structures.

Psychology research indicates that experiences such as war, persecution, or oppression remain in a collective “trauma” of a community or people. This trauma is “inherited” at the behavioral and cultural level as well as at the molecular biological level of the subsequent generations. The implications of this *“transgenerational transmission of trauma*” has been studied for various groups that have also been victims of genocide or long-term repression, such as native Australians, former black slaves in America, or Holocaust survivors [113,114,115,116].

In addition to a social transmission due to parenting and socialization, epigenetic processes are discussed in the transmission of parental PTSD. The most important epigenetic processes is DNA methylation, which means the methylation of relevant gene sections (exons) [117]. One of the affected genes is NR3C1 (Nuclear Receptor Subfamily 3 Group C Member 1) whose methylation status is responsible for both the transcription and its regulation of the glucocorticoid receptors [118]. This receptor is a relevant aspect of the HPA axis (hypothalamic pituitary adrenal axis) which is responsible—inter alia—for the glucocorticoid regulation system as complex stress response [119].

Research with women who became victims of the Tutsi genocide showed that transmission of PTSD to the offspring was associated with transmission of biological alterations of the HPA axis. There was higher methylation of the NR3C1 exon in women who were exposed to the Tutsi genocide and their children than in the non-exposed control group of the same ethnicity. Both mothers exposed to the Tutsi genocide and their children had lower levels of cortisol and glucocorticoid receptors with simultaneously higher levels of mineralcorticoid receptors. These biological alterations can promote maladaptive stress processing and, as a result, the occurrence of PTSD as well as other mental disorders in the offspring [120,121].

Indeed, the same behavior patterns that were found in people who experienced the Holocaust can be observed in Yezidis, namely feelings of insecurity, tension, worry about their children’s survival, and feelings of powerlessness and helplessness [54].

It was shown that even many generations after the traumatic experiences, the descendants suffer from increased vulnerability to various diseases. Thus, suicide rates, self-harm, and destructive behavior occur more often if actual and historical trauma are accumulating [114].

However, traditional exposure therapy is based on a western, individualistic understanding of culture and values. Therefore, it is not always effective with persons who have grown up in a collective society. Moreover, the success of the treatment largely depends on how society deals with the events [54].

Taking this into account, it is clear that there are not only consequences on the individual level. The actual extent of the events for the population can hardly be foreseen at the present time and will only reveal itself in subsequent generations. This vulnerability is promoted by the existing political, cultural, and social uncertainty and distribution of the surviving Yezidis.

Nonetheless, the current state of research on the health of Yezidi survivors, and especially Yezidi women, should also be viewed critically. Although recent research has provided first approaches and shown a high health burden, it must be noticed that there are only a few surveys, which are made more difficult by the scattering and heterogeneity of the population. Thus, some of the research has a low number of cases or only qualitative approaches. In addition, it must be taken into account that a large proportion of presented research is published by the same few research groups that might lead to a specific point of view.

## 5. Conclusions

The Yezidis in Northern Iraq are a cultural and historical “old” religious community with strong local links and deeply rooted social structures. The history of the community is marked by recurrent phases of oppression and persecution. As a result of the genocide committed by ISIS, the Yezidis once again experienced massive violence. The experiences of those affected are dependent on their gender. After a large number of the Yezidi population was murdered, Yezidi women in particular experienced massive sexual violence, such as recurring rape and sex slavery. Many Yezidi women are still in the hands of ISIS.

Research that examined the health status of surviving Yezidis who were able to escape or were freed suggests that there is a high level of health strain in this population. In addition to various infectious diseases, non-communicable diseases also play a role. The situation may be aggravated by the living situation in IDP/refugee camps: While an uncertain and precarious situation may trigger the emergence of mental diseases, the hygienic and precarious housing situation may favor the appearance of infectious and chronic diseases. In addition, the medical care is often insufficient. Research indicates a high burden of mental health problems in the surviving Yezidi women, especially PTSD and depression.

Looking at the research on transgenerational trauma, it can be assumed that the recent events in the context of genocide by ISIS revive past experiences in the history of the Yezidi community on the one hand, and consequences could be passed on to the descendants on the other hand. As well as the narrow cultural and religious rules of the community, this can be both a resource and an additional burden for those affected. In this context, the way the (Yezidi) society deals with the sexual assaults is of particular relevance. While on the one hand there is still a driving out of the events among the Yezidi genocide and the sexual assaults on Yezidi women, such as exclusion of the affected women or (forced) abortion of the ISIS fathered pregnancies, the Yezidi community is developing new strategies on dealing with this trauma on the other hand. The establishment of the described special rituals in Lalish as well as the (international) activism of affected (female) Yezidi might be seen as a newly developed coping strategy.

The actual extent of the attacks is neither predictable for the affected individuals nor for the community of the Yezidis. In addition to the development of specific, culturally sensitive therapeutic measures, long-term care and support of the affected persons and their descendants as well as the Yezidi community in dealing with the trauma seems indispensable.

It must be assumed that for this massive task, international support is needed. Both in terms of improving the present precarious situation and support of the affected women and their community in the long run, it seems to be indispensable to focus the media on these challenges to generate internationally public attention. Nominating Nadia Murad for the Nobel Peace Prize 2018 [122] and naming her “UN Goodwill Ambassador for the Dignity of Survivors of Human Trafficking” [58] as well as confirming the recent genocide [21] were important first steps with a clear international signal.

## Figures and Tables

**Table 1:** Literature search tracking sheet.

Date of Search	Database	Years Searched	Search Terms	Further Search Settings	Hits
07/11/2018	PUBMED	No restrictions	Yezidi OR Yazidi OR Ezidi	in title/abstract	16
07/11/2018	PUBMED	Since 2014	Yezidi OR Yazidi OR Ezidi AND mental	in title/abstract	10
08/11/2018	SCHOLAR	No restrictions	Yezidi OR Yazidi OR Ezidi	in title/abstract, excluding citations and patents	158
08/11/2018	DIMDI (DATAH & AMIS)	No restrictions	Yezidi OR Yazidi OR Ezidi	no restrictions	0
08/11/2018	Cochrane Library	No restrictions	Yezidi OR Yazidi OR Ezidi	in title/abstract/keyword	0

**Table 2:** Resulted literature selection.

**Persecution by ISIS and actual situation of the Yezidi community**	
**Systematic literature search**	
Research article	5
Book/Chapter	1
Working paper	1
**Additional handsearch**	
Book/Chapter	1
Research article	1
Graduate works	1
Gray literature	
Reports	1
Newspaper article	5
**Gender-specific aspects of the persecution and their consequences**	
**Systematic literature search**	
Research article	9
Graduate works	1
Comment	1
Gray literature	
Report	1
**Additional handsearch**	
Research article	3
Book/Chapter	1
Gray literature	
Book/Chapter	4
Research article	1
Reports	2
Graduate works	1
Working paper	1
**(Mental) Health of the affected women**	
**Systematic literature search**	
Reviews	3
Research article	12
Graduate works	1
Gray literature	
Working paper	1
**Additional handsearch**	
Research article	2
Gray literature	
Interview	1
Newspaper article	1
**Cultural–historical and religious context**	
**Systematic literature search**	
Research article	13
Books	10
Graduate works	3
Case reports	1
Gray literature	
Congress contribution	1
**Additional handsearch**	
Books	5
Gray literature	
Report	1
Newspaper article	1

## Supplementary Materials

The following are available online at https://www.mdpi.com/1660-4601/16/13/2435/s1, Table S1: Used literature.
