# Supplementary material for: Narrative Review: The (Mental) Health Consequences of the Northern Iraq Offensive of ISIS in 2014 for Female Yezidis
Source: Int J Environ Res Public Health. 2019 Jul 9;16(13):2435. doi: 10.3390/ijerph16132435 (PMC6651800; doi:10.3390/ijerph16132435)
Supplement: Supplementary file 1 [file ijerph-16-02435-s001.pdf]

Supplementary Material

Table S1. Used literature.

**Persecution by ISIS and actual situation of the Yezidian community**	
systematic literature search	
(Cetorelli et al. 2017c)	Research article
(Kreyenbroek and Omarkhali 2016a)	
(Spät 2018)	
(Van Schaack 2018)	
(Kizilhan 2017)	
(Al-Marashi 2017)	Book/Chapter
(Pasha 2018)	Working paper
Additional Handsearch	
(Mollica 2016a)	Book/Chapter
(Sverdlov 2017)	Research article
(Roberts 2016)	Graduate works
Gray literature	
(United Nations Human Rights 2016)	Report
Spencer, R., 2015.	Newspaper article
(Richards 2014)	
(Knights 2015)	
(Bundeszentrale für politische Bildung 2016)	
(Al Arabiya English 2017)	
Staliski, S., 2017.	
**Gender-specific aspects of the persecution and its consequences**	
systematic literature search	
(De Vido 2018)	Research article
(Nicolaus and Yuce 2017)	
(Hassen 2016)	
(Salden 2017)	
(El-Masri 2018)	
(Hechler 2016)	
(Marczak 2018)	

(Masmoudi 2018)	
(Six-Hohenbalken 2018)	
(Jelínková 2018)	Graduate works
(Mohammadi 2016)	Comment
Gray literature	
(Omer 2016)	Report
Additional Handsearch	
(Reid-Cunningham 2008)	Research article
(Kennedy 2017)	
(Buffon and Allison 2016)	
(Duarte-Herrera and Ifsits 2017)	Book/Chapter
Gray literature	
(Murad and Krajeski 2017)	Book/Chapter
(Murad 2017)	
(Schmermund 2017)	
(Joeden-Forgey 2010)	
(Buarque 2016)	Research article
(Amnesty International 2014)	Report
(United Nations Office on Drugs and Crime 2016)	
(Commandeur 2015)	Graduate works
(Guidere 2015)	Working paper
**(Health and) Mental health of the affected women**	
systematic literature search	
(Gerdau et al. 2017)	Reviews
(Kizilhan and Noll-Hussong 2017a)	
(Rodziewicz 2018)	
(Hoffman et al. 2018)	Research article
(Ibrahim et al. 2018)	
(Kizilhan 2018)	
(Jäger 2018a)	
(Arslan 2016)	
(Omarkhali 2016)	
(Erdener 2017)	
(McGee 2018)	

(Tekin et al. 2016)	
(Hillebrecht et al. 2018)	
(Jaff 2018)	
(Hosseini 2018)	
(Graeser 2018)	Graduate works
Gray literature	
(Didier 2016)	working paper
Additional Handsearch	
(Cetorelli et al. 2017a)	Research article
(Cetorelli et al. 2017b)	
Gray literature	
(Wildermann 2016)	Interview
(Hussein 2016)	Newspaper article
**Cultural-historical and religious context**	
systematic literature search	
(Fuccaro 1997)	Research article
(Allison 2008)	
(Omarkhali 2009)	
(Gündüz 2004)	
(Kreyenbroek 2008)	
(Tagay et al. 2017)	
(Allison 1998)	
(Kreyenbroek and Omarkhali 2016b)	
(Spat 2016)	
(Hutter 2018)	
(Raffaelli 2011)	
(Foltz 2017)	
(Omarkhali 2017)	
(Allison 2012)	Books
(Kreyenbroek et al. 2005)	
(Arakelova 2004)	
(Spät 2001)	
(Asatrian 1999)	
(Wießner 1984)	

(Spät 2008)	
(Allison 1996)	
(Gnau 2014)	
(Stausberg 1999)	
(Wadsworth 2017)	Graduate works
(Mollica 2016b)	
(Msall 2016)	
(Bou Khalil et al. 2018)	Case reports
Gray literature	
(Franz 1999)	Congress contribution
Additional Handsearch	
(Açikyildiz 2014)	Books
(Savucu 2016)	
(Tagay and Ortac 2016)	
(Kreyenbroek 1995)	
(Jäger 2018b)	
Gray literature	
(Wörmer and Henselmann 2016)	Report
(Asher-Shapiro 2014)	Newspaper article
